# Myocardial characterization using late enhancement photon-counting detector CT in ventricular arrhythmia: comparison with electroanatomical mapping

**DOI:** 10.1186/s13244-025-02069-4

**Published:** 2025-08-29

**Authors:** Victor Mergen, Martin F. Reiner, Konstantin Klambauer, Lukas J. Moser, Fu Guan, Corinna Brunckhorst, Firat Duru, Ernst Klotz, Thomas Flohr, Frank Ruschitzka, Robert Manka, Matthias Eberhard, Hatem Alkadhi, Ardan M. Saguner

**Affiliations:** 1 Diagnostic and Interventional Radiology, University Hospital Zurich, University of Zurich, Zurich, Switzerland; 2 Department of Cardiology, University Heart Center, University Hospital Zurich, University of Zurich, Zurich, Switzerland; 3 Center for Integrative Human Physiology, University of Zurich, Zurich, Switzerland; 4 Siemens Healthineers AG, Forchheim, Germany; 5 Center for Translational and Experimental Cardiology (CTEC), Department of Cardiology, University Hospital Zurich, University of Zurich, Zurich, Switzerland

**Keywords:** Electroanatomical mapping, Fibrosis, Photon-counting detector computed tomography, Scar, Ventricular tachycardia

## Abstract

**Objectives:**

This study aimed to assess the feasibility of left ventricular myocardial characterization in patients with ventricular arrhythmias using late enhancement (LE) photon-counting detector computed tomography (PCD-CT) scans, in comparison with invasive endocardial electroanatomical mapping (EAM).

**Materials and methods:**

This single-center retrospective observational study included 20 patients (mean age 64 ± 8 years, 4 female) who underwent PCD-CT prior to 3D endocardial uni- and bipolar EAM and radiofrequency catheter ablation (RFCA) between May 2022 and February 2024. Sixteen patients (80%) had cardiac implantable electronic devices. Twelve (60%) had ischemic and 8 (40%) had non-ischemic cardiomyopathy. Pathologic myocardial segments were defined by low-voltage electrograms < 5 mV in unipolar and < 0.5 mV in bipolar maps. Cardiac scans included LE acquisitions 5 min after contrast injection in the ECG-triggered sequential mode. Myocardial extracellular volume was computed from cardiac LE scans and visualized as polar and atlas maps (the latter depicting wall thickness) to identify pathologic segments with fibrosis and/or scar. LE scans were compared with EAM.

**Results:**

In patients with ischemic cardiomyopathy, agreement of pathologic segments on CT was good with unipolar EAM (κ = 0.655 ± 0.249), and moderate with bipolar EAM (κ = 0.547 ± 0.267). In patients with non-ischemic cardiomyopathy, agreement of pathologic segments on CT was moderate compared with unipolar (κ = 0.455 ± 0.356) and fair with bipolar EAM (κ = 0.255 ± 0.260).

**Conclusions:**

Preliminary evidence suggests that characterization of pathologic myocardial segments using LE PCD-CT scans is feasible and yields good agreement with endocardial EAM, particularly when compared with unipolar EAM and in patients with ischemic cardiomyopathy.

**Critical relevance statement:**

Characterization of pathologic left ventricular segments using myocardial extracellular volume and thickness representations from spectral late enhancement photon-counting detector CT scans indicates good agreement with unipolar endocardial electroanatomical mapping, particularly in patients with ischemic cardiomyopathy.

**Key Points:**

- Cardiac late enhancement imaging with photon-counting detector CT may enable characterization of pathologic myocardial segments in ventricular arrhythmia.
- Myocardial extracellular volume and thickness representations yield good agreement with unipolar endocardial electroanatomical mapping, particularly in patients with ischemic cardiomyopathy.
- Left ventricular myocardial characterization is feasible with late enhancement photon-counting detector CT and may complement invasive radiofrequency catheter ablations.

**Graphical Abstract:**

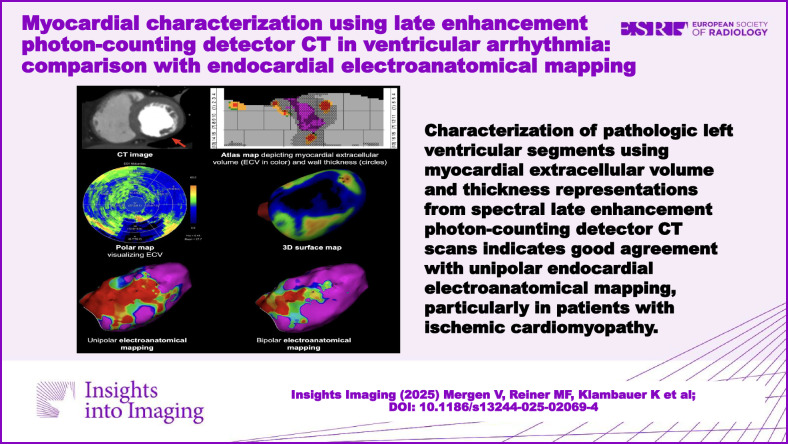

## Introduction

Ventricular arrhythmia is a leading cause of severe cardiac morbidity and mortality [1, 2]. Radiofrequency catheter ablation (RFCA) is an essential treatment strategy for patients with ventricular arrhythmias secondary to ischemic or non-ischemic cardiomyopathies, in addition to optimal medical antiarrhythmic therapy and implantable cardioverter defibrillators [1–5]. Myocardial scar is the usual substrate for arrhythmias in this patient population [2, 6]. Ventricular tachycardias (VT) are typically related to reentrant mechanisms involving the mature scar, with conduction occurring via strands of viable myocardium considered critical VT isthmuses [7]. Similarly, fibrosis plays a significant role in the arrhythmogenesis of VTs in non-ischemic cardiomyopathies [8] and fibrosis predicts ventricular arrhythmic events in these patients [9]. During RFCA, 3D electroanatomical mapping (EAM) is performed to identify scar tissue and areas that are likely to contribute to VT circuits based on low-amplitude, fractionated, and late potentials [1]. Yet, low-voltage areas may still contain viable cardiomyocytes capable of electrical conduction, and EAM is constrained by the spatial resolution of catheter dimensions and limited field depth. Furthermore, inadequate catheter-tissue contact can generate artificially low-voltage areas [1]. Cardiac imaging-complemented interventions provide accurate information on myocardial scar, resulting in higher VT-free survival rates compared with EAM and RFCA alone [10].

Cardiac magnetic resonance imaging (MRI) is considered the reference standard for visualization of myocardial scar [6, 11–15], and correlates well with EAM [13, 16, 17]. However, MRI is often not feasible in these patients due to cardiac implantable electronic devices (CIED), or the CIED cause artifacts impairing image interpretation [10, 18, 19]. Other general issues, such as claustrophobia, may arise as well.

Current guidelines recommend screening for cardiac thrombi prior to RFCA [1, 20]. Computed tomography (CT) not only facilitates the identification of cardiac thrombi using delayed enhancement scans [21] but also provides a comprehensive three-dimensional anatomic assessment of the heart for pre-procedural planning and intra-procedural guidance [20]. Moreover, previous studies have demonstrated the feasibility of myocardial scar assessment through late enhancement (LE) scans using energy-integrating detector CT (EID-CT) [22, 23]. However, these scans typically require dedicated protocols, and visual interpretation of late enhancement on EID-CTs may be challenging [22, 24]. Beyond visual interpretation, extracellular volume (ECV) maps visualize myocardial fibrosis and delineate the extent of scarring, potentially providing critical information for pre-procedural planning [20, 25]. In EID-CT, ECV maps are typically generated using an attenuation-based method that requires both unenhanced and late enhancement cardiac scans and relies on precise registration of unenhanced and LE cardiac scans [26–28].

The recently introduced photon-counting detector CT (PCD-CT) enables inherent spectral data acquisition, improved radiation dose efficiency, low electronic noise, and a high contrast-to-noise ratio of iodine-enhanced structures [29, 30]. This combination of low noise and high contrast has proven particularly beneficial for LE imaging [31–33]. Importantly, spectral cardiac scans can now be performed at the highest temporal resolution, an advancement not previously achievable with EID-CT [29, 34]. As a result, ECV values can be derived solely from the spectral LE scan, eliminating the need for registration between unenhanced and LE scans, and thereby improving the robustness of the method compared with conventional EID-CT [31, 32, 35].

The aim of the study was to assess the feasibility of left ventricular myocardial characterization in patients with ventricular arrhythmias using LE PCD-CT scans in comparison with invasive endocardial EAM.

## Materials and methods

### Patients

Consecutive patients who underwent routine pre-procedural CT with PCD-CT prior to endocardial EAM and RFCA of the left ventricle for ventricular arrhythmias between May 2022 and February 2024 were retrospectively identified. Patients with incomplete EAM of the left ventricle were excluded. All patients provided written informed consent to allow the inclusion of their anonymized data in retrospective analyses. The study complies with the Declaration of Helsinki and was approved by the local ethics committee (BASEC Nr. 2021-00343).

### CT data acquisition and image reconstruction

Scans were acquired on a dual-source PCD-CT system (NAEOTOM Alpha; Siemens Healthineers; VB10 version) equipped with two cadmium telluride detectors. The routine scan protocol comprises a non-contrast cardiac scan, a coronary CT angiography, and a cardiac LE scan. All cardiac scans were acquired in the ECG-triggered, sequential, spectral mode (QuantumPlus, Siemens). The coronary CT angiography was initiated after the injection of a weight-based volume of iodinated contrast medium (72–98 mL, iopromide, Ultravist 370 mg I/mL; Bayer Healthcare), followed by a saline chaser (20 mL, NaCl 0.9%) applying a weight-based flow rate (5.0–6.0 mL/s). For coronary CT angiography, a variable ECG-triggering depending on the heart rate was used. The cardiac LE scan was acquired 5 min after the initiation of the single administration of contrast medium, using a fixed ECG-triggering delay of 280 ms after the RR-peak. Patients did not obtain any medication prior to the scan. Further parameters of the cardiac LE scan are detailed in Table 1.

**Table 1:** Scan parameters and scan doses

	Late enhancement CT
Scan parameters	
Tube voltage (kVp)	140
Image quality level	80
Tube current time product (mAs)	43 ± 21
Detector collimation (mm)	144 × 0.4
Gantry rotation time (s)	0.25
Radiation dose estimates	
Volume CT dose index (mGy)	9.2 ± 3.2
Dose length product (mGy*cm)	131 ± 53
Size-specific dose estimate (mGy)	11.6 ± 2.5

Data are expressed as mean ± SD or absolute values

Coronary CT angiographies and cardiac LE scans were reconstructed as virtual monoenergetic images at 65 keV. In addition, cardiac LE scans were reconstructed as iodine images. All images had a slice thickness of 1.5 mm, an increment of 1.0 mm, used the Qr40 kernel and applied quantum iterative reconstruction (QIR) strength 3. Field-of-view was 200 × 200 mm^2^ and matrix size 512 × 512 pixels. The hematocrit (in L/L) required for the ECV computation was retrieved from the medical records.

#### Overlay images, polar and atlas maps

ECV maps were generated using a dedicated research software (CT Cardiac Functional Analysis Frontier, version 3.0; Siemens Healthineers AG), as previously described [31]. Virtual monoenergetic images from the coronary CT angiographies and the LE scans, as well as the iodine maps from the LE scans, served as input series. Quantitative ECV values were derived exclusively from the iodine maps of the LE scans. The resulting ECV values were subsequently presented as color-coded polar maps, as overlays onto short and long-axis views of the coronary CT angiography, and as 3D surface overlays.

**Fig. 1:**
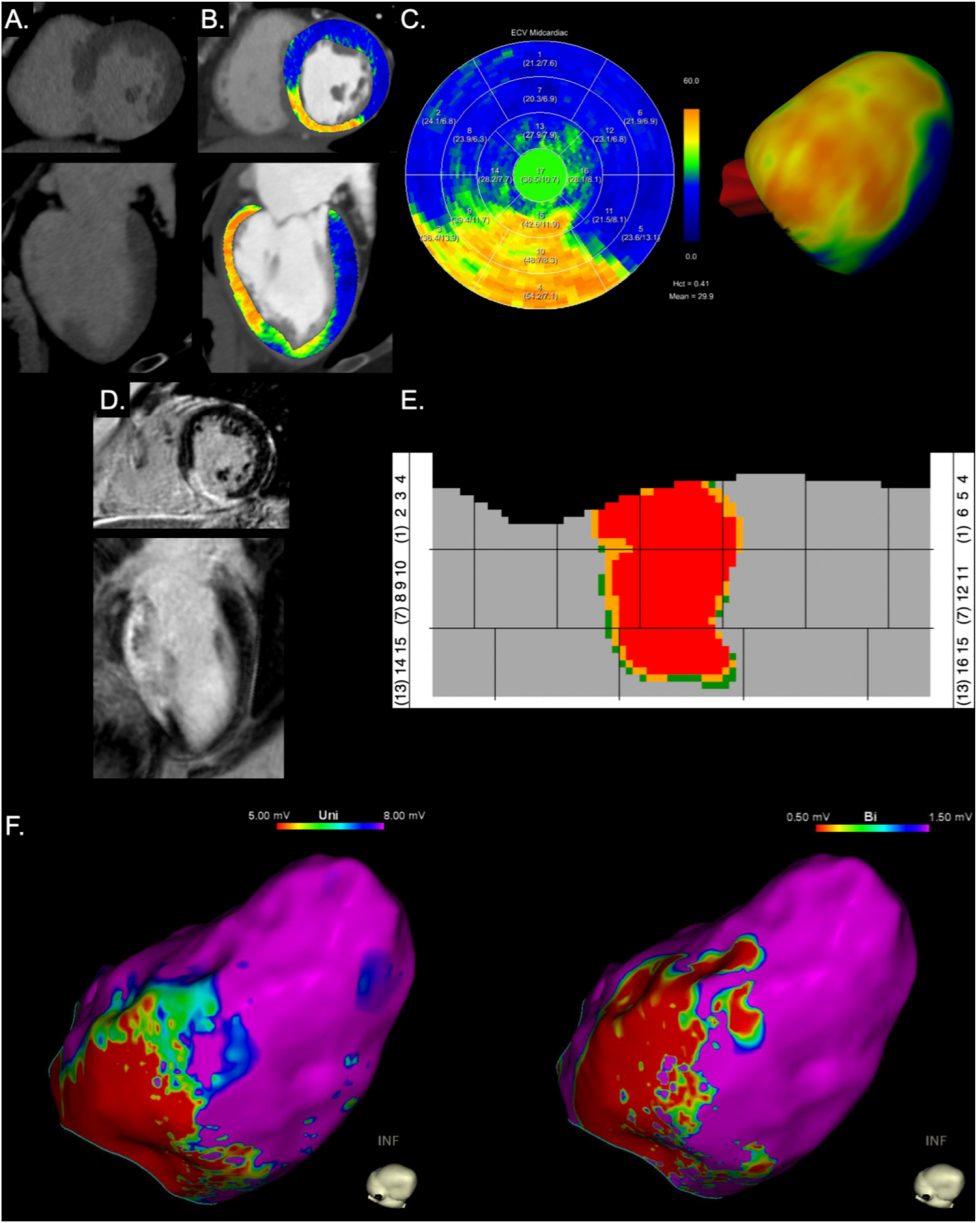
Representative images of the left ventricle of a 62-year-old female patient with ischemic cardiomyopathy. Virtual monoenergetic images (at 65 keV) of cardiac late enhancement photon-counting detector CT show iodine enhancement of the inferior wall of the left ventricle (**A**). Corresponding high extracellular volume (ECV) is demonstrated in coronary CT angiography with an overlay visualizing ECV (**B**), as well as in ECV polar maps (**C**) and atlas maps (**E**). Prior cardiac MR imaging shows identical extent of late gadolinium enhancement (**D**) to the ECV elevation observed in late enhancement CT. Endocardial electroanatomic mapping (EAM) (**F**) shows myocardial low-voltage areas of the inferior/inferolateral basal and inferio/inferoseptal midventricular segments in unipolar (< 5.0 mV) and bipolar (< 0.5 mV) EAM

**Fig. 2:**
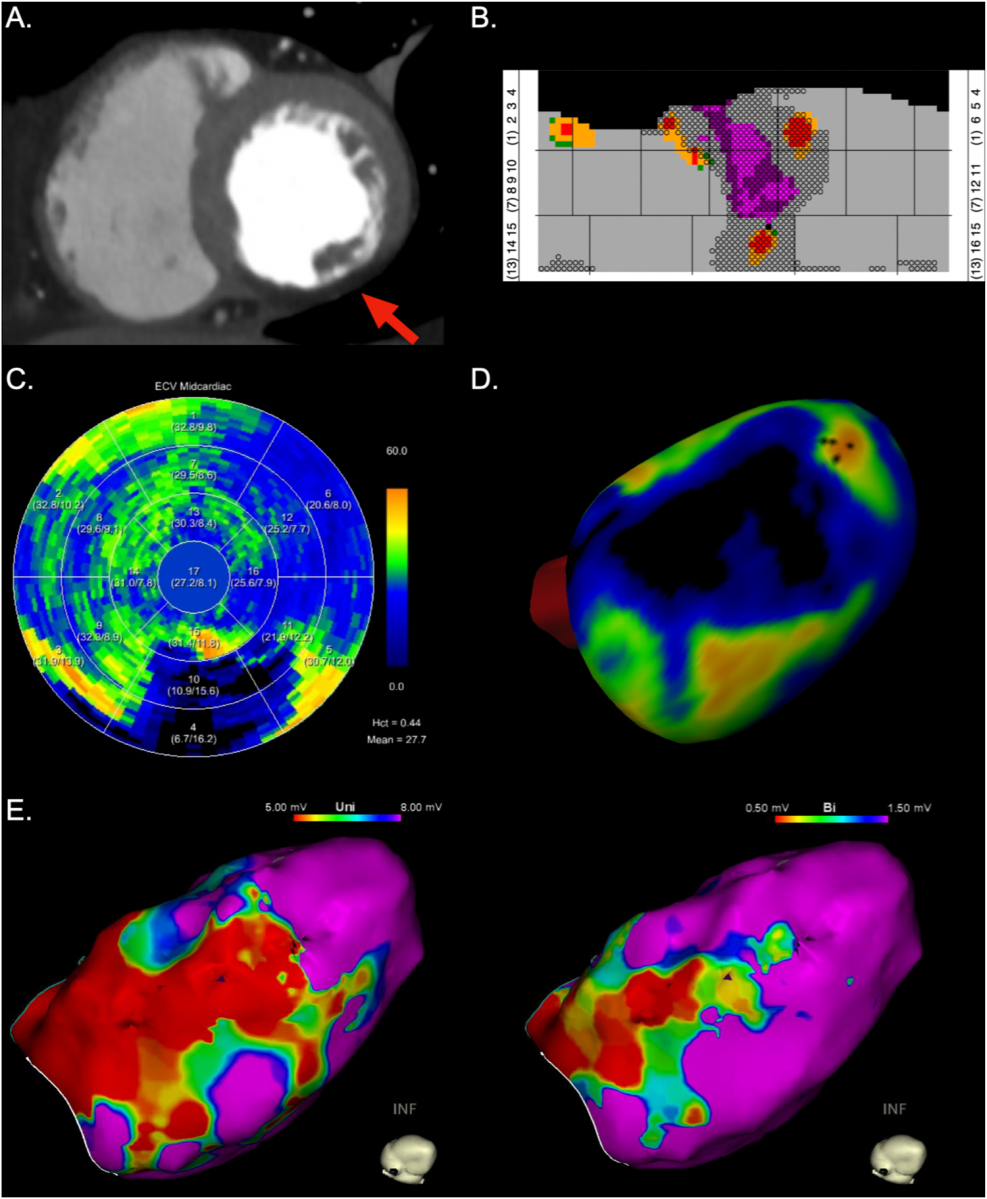
Representative images of the left ventricle of an 82-year-old male patient with ischemic cardiomyopathy. Coronary CT angiography shows myocardial thinning with fatty metaplasia of the inferior wall of the left ventricle (red arrow, **A**), which is visualized on the atlas map (pink color indicating fatty metaplasia and circles indicating myocardial thinning, **B**). Adjacent to myocardial thinning, fibrosis reflected by elevated extracellular volume (ECV) can be observed (**B**–**D**). Endocardial electroanatomic mapping (EAM) (**E**) shows corresponding myocardial low-voltage areas of the septal/inferior/inferolateral basal, the inferseptal/inferior/inferolateral midventricular and inferior apical segments in unipolar (< 5.0 mV) EAM and the septal/inferior basal and inferior midventricular segments in bipolar (< 0.5 mV) EAM

Additional high-resolution atlas maps were created from the ECV values with the support of a consultant of Siemens (E.K.) [36]. Atlas maps provide two-dimensional planar representations of myocardial ECV, with the myocardium depicted as a rectangle cut open anteriorly at segments 1, 7, and 13. These maps provide a condensed overview of relevant information, including information about myocardial wall thickness. ECV is classified with a color-coded triple threshold: red for ECV > 45%, orange for ECV > 40%, and green for ECV > 35%, as previously shown [36] (Figs. 1, 2). Additionally, areas with suspicious ECV values, likely caused by metal artifacts or fatty metaplasia, are labeled in dark magenta (ECV > 65% or < 10%) or light magenta (ECV > 75% or < 0%). Finally, areas with an estimated wall thickness of below 5 mm, as previously described [22], are marked with circles (Fig. 2). More details about the rationale and technique to create these atlas maps are provided in the Supplementary Material.

### CT image interpretation

Coronary CT angiographies and cardiac LE scans were displayed as two-chamber short-axis, two-chamber long-axis, and four-chamber long-axis CT reformats alongside ECV polar maps, and atlas maps.

Two independent readers (V.M. and K.K.) assessed sixteen myocardial segments according to the American Heart Association polar map for evidence of injured myocardium. Segment 17, the apex, was excluded, as this segment cannot be adequately judged by EAM. Both readers were informed whether the patient had ischemic or non-ischemic cardiomyopathy, but were blinded to the results of endocardial EAM. One reader (V.M.) additionally graded images using a three-point visual scale, as myocardial segments can be affected by beam hardening artifacts from adjacent leads of CIEDs: 3 = excellent, no beam hardening artifacts; 2 = sufficient, artifacts are visible but do not impair confident assessment, and 1 = insufficient, beam hardening prohibits segment assessment.

A myocardial segment was deemed pathologic—exhibiting myocardial fibrosis or scarring—if it demonstrated regions with visually elevated ECV or myocardial thinning (< 5 mm) [22], potentially accompanied by fatty metaplasia or aneurysmal dilatation. Notably, all patterns could coexist within a single segment. ECV or atlas maps did not permit a reliable distinction between subendocardial, mesocardial, and epicardial patterns of fibrosis. All cases were read, and segments with insufficient image quality were excluded from further analysis.

### Endocardial electroanatomical mapping and evaluation

3D endocardial three-dimensional (3D) EAM of the left ventricle was performed with a multipolar catheter (PentaRay, Biosense Webster Inc.) and/or a radiofrequency ablation catheter (Smart Touch ST/SF 3.5 mm, Biosense Webster) using CARTO V3 (Biosense Webster). Mapping was performed during sinus rhythm. Dense scar was defined by low-voltage electrograms < 5 mV in unipolar and < 0.5 mV in bipolar mapping. Left ventricular segments affected by myocardial scar were identified by consensus reading of two cardiologists (M.F.R. and A.M.S.). Activation, pace and voltage maps were used to guide RFCA, which was performed in power‐controlled mode, power 25 to 45 W, irrigation 17 to 30 mL/min, and temperature limit 43 °C.

### Statistical analysis

Variables are presented as means ± SDs when normally distributed and as medians and interquartile ranges for skewed distribution. Categorical variables are reported as counts and percentages. Segments with insufficient image quality were excluded from further analysis. Interreader agreement and agreement between CT and EAM were evaluated using Cohen’s unweighted κ statistic for binary classifications (from 0 to 1.00; with 1.00 to 0.80 indicating very good agreement; from 0.80 to 0.60, good; from 0.60 to 0.40, moderate; from 0.40 to 0.20, fair; and below 0.20, poor agreement) [22]. CT interreader agreement was calculated using the readings of both readers. Subsequently, the readings of reader 1 were used for comparison with EAM. Agreement between CT and EAM was calculated per segment and then averaged over segments as previously described [22]. Analyses were performed using R statistical software (R, version 4.4.2; R Foundation).

## Results

### Patient population

**Fig. 3:**
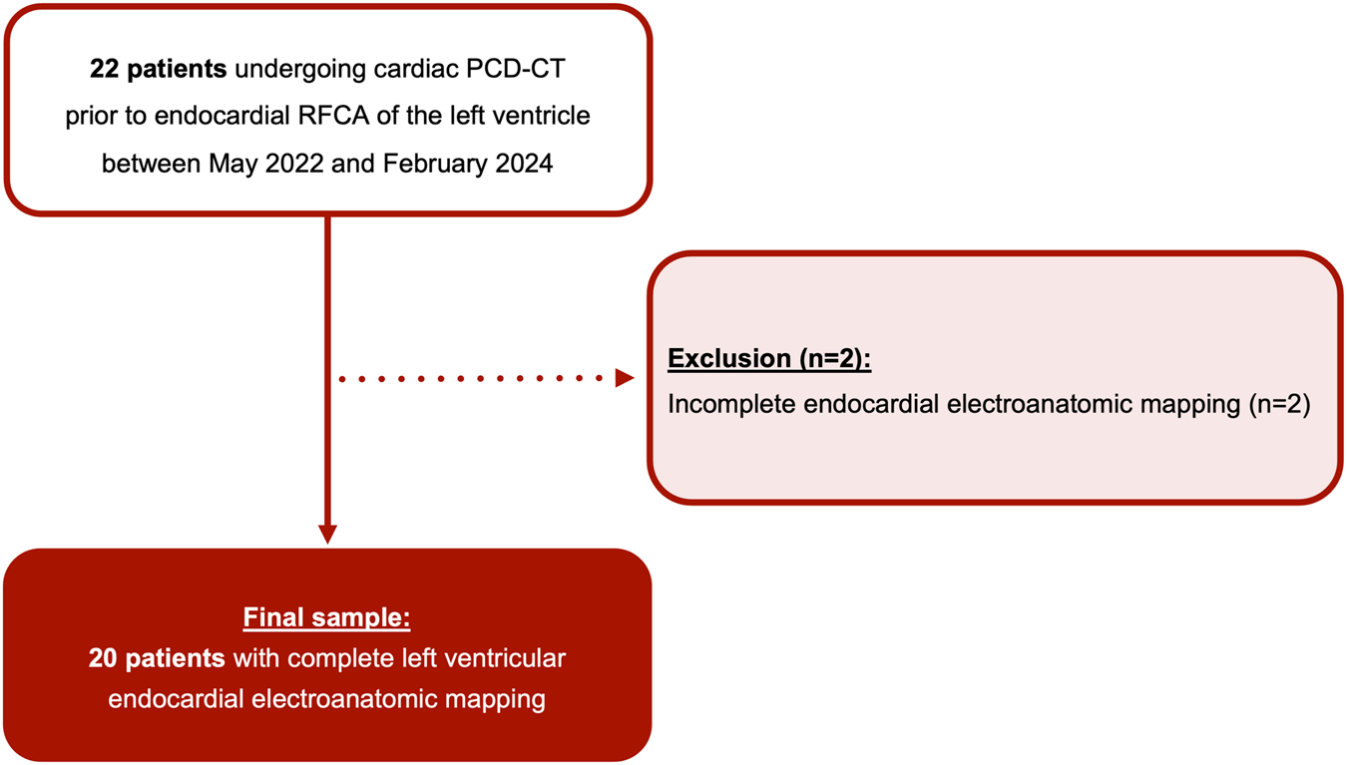
Study flowchart. PCD-CT, photon-counting detector CT; RFCA, radiofrequency catheter ablation

Of the 22 patients who underwent PCD-CT including LE imaging prior to endocardial RFCA of the left ventricle, two were excluded because of incomplete left ventricular EAM (Fig. 3). Finally, 20 patients were included (4 [20%] female; mean age of all patients 64 ± 8 years; mean body mass index 28.1 ± 4.4 kg/m^2^). Of the 20 patients, 12 (60%) had ischemic cardiomyopathy, and 10/12 (83%) had CIEDs (2 cardiac resynchronization therapy defibrillator devices and 8 implantable cardioverter defibrillators). The remaining 8 (40%) had non-ischemic cardiomyopathy, and 6/8 (75%) had a CIED (2 cardiac resynchronization therapy defibrillator devices and 4 implantable cardioverter defibrillators). In patients with non-ischemic cardiomyopathies, 4 (50%) patients had dilated cardiomyopathy, 3 (38%) patients had non-dilated left ventricular cardiomyopathy, and one (12%) patient had post-traumatic cardiomyopathy after perforation of the left ventricle with consecutive surgical repair. Fourteen out of 20 (70%) patients received ablation of VT, and 6 (30%) patients received ablation of ventricular ectopy.

Median heart rate during acquisition of the cardiac LE scan was 67 beats per minute (interquartile range (IQR), 58–73). Mean hematocrit was 0.445 ± 0.046 L/L. The hematocrit was obtained on the same day as CT in 9/20 patients (45%), within 1 day in 10/20 patients (50%), and within 3 days in one patient (5%). Table 2 details the baseline characteristics of the included patients.

**Table 2:** Baseline characteristics

Patient characteristics	All patients (*n* = 20)
Sex	
Male	16 (80%)
Female	4 (20%)
Age (years)	64 ± 8
Body mass index (kg/m^2^)	28.1 ± 4.4
Heart rate during the scans (bpm)*	67 (58–73)
Mean hematocrit (L/L)	0.445 ± 0.046
Ischemic cardiomyopathies	12 (60%)
Cardiac implantable electronic device	10 (83%)
Non-ischemic cardiomyopathies	8 (40%)
Dilated cardiomyopathy	4 (50%)
Non-dilated left ventricular cardiomyopathy	3 (38%)
Post-traumatic cardiomyopathy	1 (12%)
Cardiac implantable electronic device	6 (75%)
Endocardial radiofrequency ablation	
Ablation of ventricular tachycardia	14 (70%)
Ablation of ventricular ectopy	6 (30%)

Data are expressed as mean ± SD or proportion of patients with percentages in parentheses

* Data are medians, with IQRs in parentheses

### Comparison between cardiac PCD-CT and endocardial EAM in ischemic cardiomyopathy

CT and EAM were performed on the same day in 6/20 patients (30%), within 1 day in 13/20 patients (65%), and within 3 days in one patient (5%). In the 12 patients with ischemic heart disease, 192 (16 × 12) myocardial segments were assessed. In CT, 5/192 segments (3%) were excluded due to insufficient image quality (score 1) because of severe beam hardening artifacts from adjacent leads of CIEDs; two segments were inferoseptal basal (2/5, 40%), two were inferoseptal midventricular (2/5, 40%), and one segment was inferior apical (1/5, 20%). 28/192 segments (15%) had sufficient quality (score 2), and 159/192 segments (83%) were rated as having excellent quality (score 3).

**Fig. 4:**
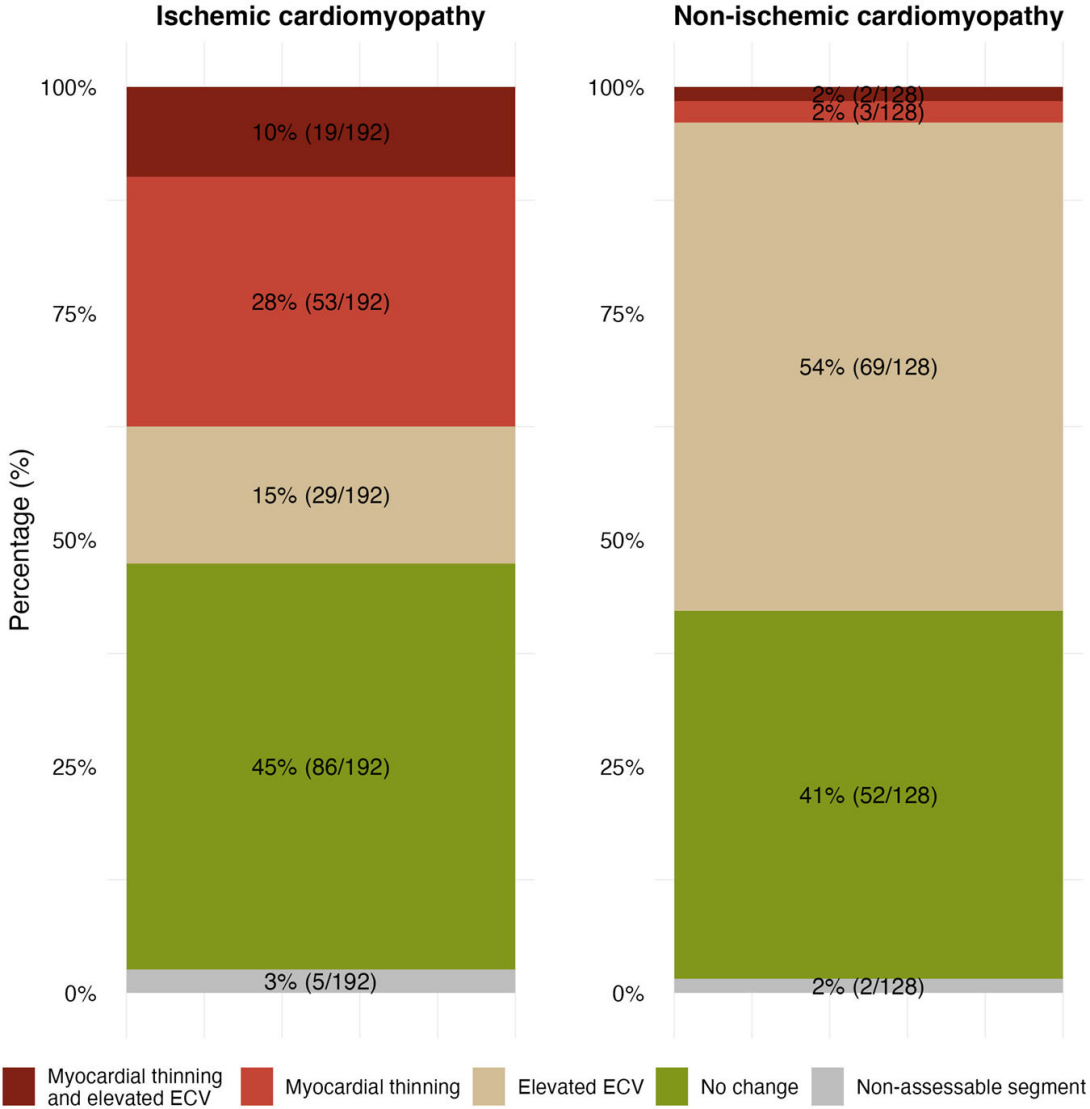
Distribution of CT changes observed in myocardial segments deemed pathologic. ECV, extracellular volume

Interreader agreement of segments deemed pathologic on CT was very good (κ = 0.875 ± 0.169). CT identified a median of 8 (IQR, 7–11) pathologic segments per patient. Most common segments with pathological myocardium were inferior basal (9/12 segments, 75%), inferior apical (8/11 segments, 73%), inferoseptal basal (7/10 segments, 70%), inferolateral basal and lateral apical (both, 8/12 segments, 67%). Predominant CT changes in ischemic cardiomyopathy were segments with myocardial thinning (53/192 segments, 28%), followed by elevated ECV (29/192 segments, 15%), and segments with concomitant myocardial thinning and elevated ECV (19/192 segments, 10%) (Fig. 4).

Unipolar and bipolar EAM identified a median of 9 (IQR, 6–11) and 8 (IQR, 5–9) low-voltage segments, respectively. Agreement was good between CT and unipolar (κ = 0.655 ± 0.249) and moderate between CT and bipolar EAM (κ = 0.547 ± 0.267). Representative images of PCD-CT and invasive EAM of the left ventricle of patients with ischemic cardiomyopathy are presented in Figs. 1 and 2.

### Comparison between cardiac PCD-CT and endocardial EAM in non-ischemic cardiomyopathy

In the eight patients with non-ischemic cardiomyopathy, 128 (16 × 8) myocardial segments were assessed. On CT, 2/128 segments (2%) were excluded due to insufficient image quality (score 1) because of severe beam hardening artifacts from adjacent leads of CIEDs. One segment was anterior basal (1/2, 50%), and one segment was inferoseptal basal (1/2, 50%). 27/128 segments (21%) had sufficient quality (score 2), and 99/128 segments (77%) were rated as excellent (score 3).

Interreader agreement of segments deemed pathologic on CT was very good (κ = 0.830 ± 197). CT identified a median of 9 (IQR, 4–16) pathologic segments per patient. Most common segments with pathological myocardium were anterior midventricular (7/8 segments, 88%), anterior apical (6/8 segments, 75%), inferoseptal and inferior basal (both, 5/7 segments, 71%). Predominant CT changes in non-ischemic were segments with elevated ECV (69/128 segments, 54%). Only the patient with post-traumatic post-surgical myocardial injury showed thinned myocardial segments (5 segments) (Fig. 4).

**Fig. 5:**
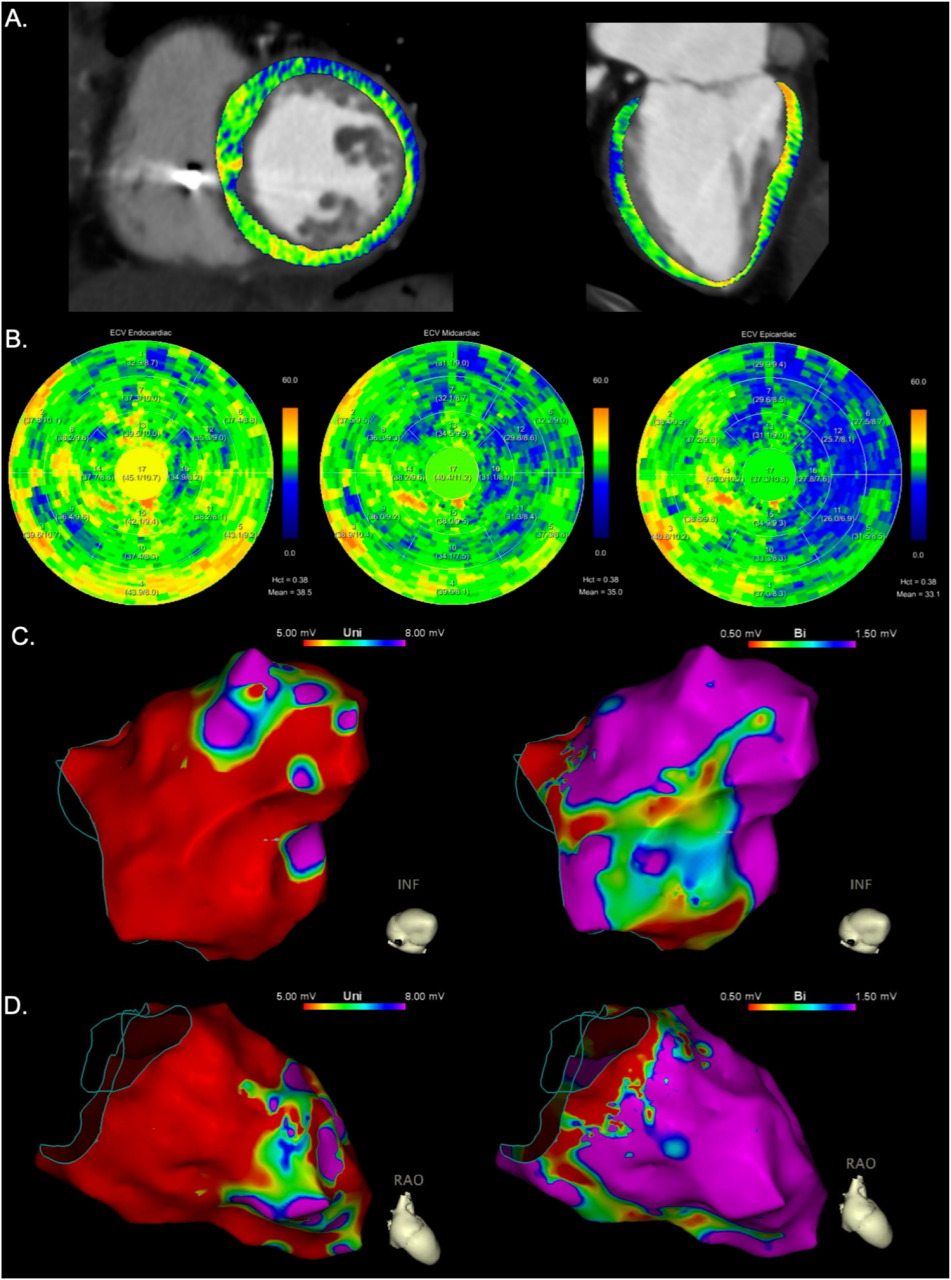
Representative images of the left ventricle of a 69-year-old female patient with dilatative cardiomyopathy. Coronary CT angiography with an overlay visualizing the extracellular volume (ECV) (**A**) and ECV polar maps (**B**) indicates diffuse fibrosis of the whole myocardium of the left ventricle. Polar maps suggest a fibrosis gradient from endocardial (inner 10% to 50% of the myocardium), to midcardiac (inner 25% to outer 25%), and epicardial (50% to outer 10%) layer. Endocardial electroanatomic mapping (EAM) (**C**, **D**) shows myocardial low-voltage areas of all left ventricular segments in unipolar (< 5.0 mV) EAM and low-voltage areas in the basal segments, as well as in the inferior segments in bipolar (< 0.5 mV) EAM

Unipolar and bipolar EAM identified a median of 6 (IQR, 6–14) and 4 (IQR, 3–5) low-voltage segments, respectively. Agreement was moderate between CT and unipolar EAM (κ = 0.455 ± 0.356) and fair between CT and bipolar EAM (κ = 0.255 ± 0.260). Representative images of PCD-CT and invasive EAM of the left ventricle of a patient with dilated cardiomyopathy are presented in Fig. 5.

## Discussion

Characterization of pathologic myocardial segments using LE scans from PCD-CT may complement EAM and guide RFCA for ventricular arrhythmias, particularly in patients in whom MRI is not feasible or is associated with relevant artifacts. In this retrospective, observational study of patients with ischemic and non-ischemic cardiomyopathy undergoing PCD-CT prior to RFCA for ventricular arrhythmia, we assessed the feasibility of left ventricular myocardial imaging using LE PCD-CT in comparison with invasive endocardial EAM. In addition, we introduced atlas maps for the visualization of chronic myocardial scars, including both information about myocardial fibrosis and myocardial thinning, which have the potential to further enhance LE CT image interpretation. Our preliminary evidence suggests that left ventricular myocardial characterization in patients with ventricular arrhythmia is feasible with a good agreement of pathologic myocardial segments with unipolar EAM, particularly in patients with ischemic cardiomyopathy. Agreement was lower in patients with non-ischemic cardiomyopathies and with bipolar EAM.

While MRI remains widely used for myocardial substrate characterization and guidance in RFCA procedures, cardiac CT has emerged as an increasingly valuable alternative [20]. Compared with MRI, cardiac CT offers shorter imaging acquisition times, requires only short breath holds, provides higher spatial and temporal resolution, and claustrophobia is rarely a constraint. A previous study by Esposito et al reported a good agreement between scars identified on pre-procedural conventional EID-CT and low-voltage segments on EAM (κ = 0.536) [22]. Similarly, a study by Carbucicchio et al found a strong correlation of myocardial fibrosis determined by conventional CT and EAM in patients with ischemic and non-ischemic cardiomyopathies (κ = 0.796–1.0) [23]. In addition, Englert et al indicated that a conventional CT-guided RFCA procedure significantly reduced the rate of VT recurrence compared with procedures without CT guidance, highlighting the relevance of pre-procedural cardiac CT in improving patient outcomes [37]. All three studies identified myocardial fibrosis as areas appearing hyperdense on cardiac LE images compared with healthy myocardium [22, 23, 37]. Beyond relying solely on attenuation differences to identify areas of myocardial fibrosis, quantifying and subsequently visualizing myocardial ECV with color coding offers the potential to enhance the detection and characterization of myocardial fibrosis. Several studies found strong correlations between myocardial ECV calculated from LE cardiac CT and MRI [32, 38–40].

Conventional EID-CT calculates ECV using an attenuation-based method. Attenuation differences between myocardial LE scans and unenhanced scans identify areas of high attenuation, which indicate iodine accumulation and thereby reflect elevated ECV [24, 31]. However, the several-minute delay between unenhanced and LE scans, along with potential variations in breath-hold depths and differences in heart rates, increases the risk of misaligned artifacts when generating ECV maps [31]. The novel PCD-CT used in this study offers several advantages over conventional EID-CT and other non-invasive imaging modalities. Notably, PCD-CT enables cardiac imaging with spectral resolution in dual-source mode, achieving a high temporal resolution of 66 ms [29]. This is feasible because photon-counting detector performs the spectral separation at the detector level, unlike in EID-CT, where obtaining spectral information is only feasible through adjustments to the acquisition modes, typically at the expense of temporal resolution [24, 41]. Importantly, PCD-CT enables ECV calculation based solely on iodine maps reconstructed from a single LE scan acquired at a high temporal resolution [35]. This approach substantially enhances the robustness of ECV computation, which is of particular importance in patients with metallic CIEDs [31]. Despite 80% of patients in our study having a CIED, only 3% of myocardial segments in patients with ischemic cardiomyopathy and 2% in those with non-ischemic cardiomyopathy were classified as non-diagnostic.

In ischemic cardiomyopathy, the predominant changes indicating myocardial injury were myocardial thinning, observed in 28% of segments, elevated ECV in 15% of segments, and the combination of both in 10% of segments. However, myocardial thinning is not depicted on myocardial ECV polar maps. Hence, we developed an in-house representation using atlas maps that combine both color-coded myocardial ECV visualization and geometric forms to indicate myocardial thinning. The key difference between these visualization methods is that polar maps display ECV values but do not show myocardial thinning, while atlas maps combine both ECV and wall thickness information. Based on our preliminary experience, we believe that such atlas maps illustrating both elevated ECV and myocardial thinning offer a more intuitive and comprehensive evaluation of chronic myocardial scars.

While identification of myocardial scar and critical VT isthmuses by EAM correlates well with cardiac CT and MRI [16, 17, 42], delineation of pathological myocardium using EAM may be limited due to insufficient catheter contact resulting in low-voltage electrograms and low spatial resolution due to high interelectrode distance of mapping catheters [1, 10]. Furthermore, scar identification by EAM is prone to reduced sensitivity for far-field signals originating from the midmyocardium and epicardium [43]. A previous study by Wijnmaalen et al in patients with ischemic heart disease found that bipolar EAM incompletely detected non-transmural scars compared with MRI [43]. Similarly to non-transmural scar in ischemic heart disease, intramural/epicardial patchy scar—the dominant form in non-ischemic heart disease [44, 45]—may be underestimated by bipolar voltage mapping and is more accurately detected using unipolar mapping [10]. This is in line with our results showing that unipolar electrograms identified a higher number of pathological left ventricular segments, resulting in better correlation with PCD-CT in both patients with ischemic and non-ischemic cardiomyopathies.

The following limitations merit consideration. First, this retrospective, single-center study included a limited number of patients, and the etiology of cardiomyopathies was heterogeneous. Second, cardiac late enhancement scans were acquired using only the ECG-triggered, sequential, spectral mode. Third, a recently developed nonrigid registration software designed to reduce stair-step artifacts [46], which may be particularly useful in patients with arrhythmias, was not evaluated. Notably, no scan had to be excluded because of pulsation artifacts or artifacts caused by arrhythmia. Fourth, ECV evaluation was performed using a prototype software and an in-house developed atlas map representation. However, we are confident that the ECV calculation software will soon become commercially available. Once available, the impact of intra-procedural image guidance using ECV visualization should be systematically evaluated. Fifth, the pathology-specific discriminatory capacity of ECV was not evaluated, as a direct comparison of CT findings and another cardiac imaging modality was not feasible due to the absence of routine pre-procedural cardiac CT and MRI in the same patients. Nevertheless, previous studies have demonstrated a high concordance between LE imaging using PCD-CT and MRI for myocardial tissue characterization [32, 36]. Finally, patients underwent endocardial EAM of the left ventricle, and the results cannot directly be extrapolated to epicardial mapping or right ventricular mapping [1, 45].

In conclusion, preliminary evidence suggests that characterization of pathologic myocardial segments using LE PCD-CT scans is feasible and yields good agreement with endocardial EAM, particularly when compared with unipolar EAM and in patients with ischemic cardiomyopathy. Newly introduced atlas maps illustrating both areas with elevated ECV and myocardial thinning offer an intuitive and comprehensive evaluation of pathologic myocardial segments. Thus, LE imaging with PCD-CT has the potential to be used for pre-procedural substrate characterization and guidance of catheter ablation in patients with ventricular arrhythmias, which needs to be confirmed in future studies with larger patient cohorts.

## Supplementary information

Electronic Supplementary Material

## Data Availability

Data generated or analyzed during the study are available from the corresponding author upon request.

## Abbreviations

CIED: Cardiac implantable electronic device
EAM: Electroanatomical mapping
ECV: Extracellular volume
EID-CT: Energy-integrating detector CT
LE: Late enhancement
PCD-CT: Photon-counting detector CT
RFCA: Radiofrequency catheter ablation
VT: Ventricular tachycardia
