# Supplementary material for: Myocardial characterization using late enhancement photon-counting detector CT in ventricular arrhythmia: comparison with electroanatomical mapping
Source: Insights Imaging. 2025 Aug 29;16:187. doi: 10.1186/s13244-025-02069-4 (PMC12397451; doi:10.1186/s13244-025-02069-4)
Supplement: Supplementary file 1 — Electronic Supplementary Material [file 13244_2025_2069_MOESM1_ESM.pdf]

# Myocardial Characterization using Late Enhancement Photon-Counting Detector CT in Ventricular Arrhythmia: Comparison with Electroanatomical Mapping

# ELECTRONIC SUPPLEMENTARY MATERIAL

## Detailed Methods

*Creation of atlas maps*

A major challenge of myocardial late enhancement imaging with CT is the low iodine concentration present in this quasi-equilibrium state, particularly if routine doses of iodinated contrast medium (27 to 36 g of iodine) are administered as in this study. Compared with cardiac magnetic resonance imaging (MRI), where 0.2 mmol/kg of gadolinium-based contrast medium are injected for the late gadolinium enhancement (LGE) scans, attenuation differences obtained in CT are considerably lower. CT provides a complete 3D analysis of the whole heart with substantially higher spatial sampling. In this study, the typical voxel size of reconstructions of the late enhancement scans was 0.4*0.4*1.0 mm resulting in well over 500,000 voxels available for analysis. Scans performed with the photon-counting detector CT (PCD-CT) are practically free of electronic noise contributions. However, random statistical fluctuations inherent when measuring photons are unavoidable. These fluctuations result in a relatively low signal-to-noise ratio (SNR) in iodine images reconstructed from the cardiac late enhancement scans at this resolution level. Nevertheless, the random distribution of noise allows a substantial reduction through averaging over a sufficient number of voxels. Recent work has shown that averaging over suitable anatomical sub-compartments (AHA 16 segments split into endo and epicardium

layers) provides sufficient precision to reliably measure regional variations down to 1% extracellular volume (ECV) [1].

To assess the extent of focal lesions, we employed the concept to sample compartments that were large enough to reduce noise yet small enough to resolve the lesions. These compartments are constructed from the automatic heart-model of the CFA research software by subdividing the heart along the long axis into 34 short axis layers (compared to 3 in AHA16) and into 72 angular segments (compared to 6 or 4 in AHA16). Voxels are collected by a raytracing algorithm originating from the long axis that allows to separately collect data for the endocardium, the mid-wall and the epicardium. These data are presented per layer with a continuous color lookup table in the research software in the form of “AHA similar” polar maps. Additionally, the mean ECV values and the corresponding number of voxels for each of the 3*34*72 segments are exported in platform independent csv format. The choice of 34*72 was motivated by practical considerations and the aim to roughly adjust the segment size to the size of a typical LGE cardiac MRI voxel. For a heart of 7 cm height and 5 cm diameter each segment spans about 2*2 $mm^2$ on the surface and with a wall thickness of roughly one centimeter it covers a volume of 20 $mm^3$.

In PCD-CT, late enhancement imaging and ECV determination are practically equivalent. ECV has the benefit that objective thresholds can be used for segmentation if the SNR is raised. To do so, we interpreted each of the three layers as a 34*72 matrix, which was arbitrarily cut open anteriorly (center of segments AHA 1,7,13). Each layer was processed to remove non-connected speckle noise present in the polar maps using a 5 by 5 median filtration. A filter size of 5 achieves a noticeable noise reduction while fully retaining lesions of about 1 cm diameter on the surface. The filtered data were then subjected to thresholding using 45%, 40% and

35% ECV as markers of a descending probability for the presence of a focal lesion. The thresholds were selected from first principles, where values below 30% are most likely non-pathological and values above 50% are almost certainly pathological, and from previous work on patients with ischemic scars.

Considering metal artifacts in PCD-CT, a monochromatic beam hardening correction reduces their magnitude considerably, but remaining level changes might still influence quantification. We therefore added a warning marker to the atlas maps if ECV values were suspiciously high or low, since beam hardening effects can be positive or negative. If ECV values were higher than 65% or lower than 10%, elements were marked with dark magenta, and if ECV values were higher than 75% or lower than 0% (definitely non-physiological), elements were marked with bright magenta.

As a final visual aid, we estimated the regional wall thickness using the available layer data. Accurately representing the true wall thickness would require calculating the orthogonal distance from the epicardial to the endocardial borders. This complex task requires access to the full surface mesh, which was unfortunately not available. Therefore, we opted for an approximate surrogate. Volume data was sampled by orthogonal raytracing to the long axis for basal and midventricular slabs (a cylinder coordinate system) and by hemispherical (polar) raytracing for the apical portion. Since the endo- and epicardial layers are precisely separated at 50% of the traversing ray, the distance along the ray can be derived using the volume difference between the layers and the slab height. Elements with an estimated thickness of less than 5 mm were marked with a circle in the atlas. This feature provides guidance during evaluation, but the final decision for an AHA segment should always be based on the CT reformats.

Filtering and creation of atlas maps were done with offline in-house code programmed in R and results were generated as pdf files.

## References

1 Patel KP, Scully PR, Saberwal B et al (2024) Regional Distribution of Extracellular Volume Quantified by Cardiac CT in Aortic Stenosis: Insights Into Disease Mechanisms and Impact on Outcomes. Circ Cardiovasc Imaging 17:e015996
